# Observations on the Pathology and Treatment of Neuralgia

**Published:** 1843-12

**Authors:** Thomas Barbour


### WESTERN JOURNAL

OF

### MEDICINE AND SURGERY

---

DECEMBER, 1843.

---

Art. I.—*Observations on the Pathology and Treatment of Neuralgia.* Communicated to the Medical Society of Columbia, Tennessee. By Thomas Barbour, M.D. [Published by request of the Society.]

In the whole circle of medical science, there is no subject that is involved in so much obscurity as that of the true pathology of neuralgia; and none that is more worthy of our attentive investigation, with the view to determine, if possible, its seat, its essential nature, and the best method of treatment.

As it should be the paramount object of every one who is engaged in the pursuit of knowledge, to discover and exhibit truth, rather than attempt to establish some preconceived opinion, it is my design, in the following brief communication, not to assume, in the spirit of dogmatism, any particular position, but merely to present the results of my observation and experience, for the consideration of my professional brethren—with the hope that they may serve *in some degree* to elucidate the pathology, and improve the treatment of neuralgic affections.

I propose to discuss in an impartial manner, the question which has been selected for debate, namely: Is neuralgia an idiopathic affection of the nervous centres and branches, or is it sympathetic of remote organic irritation?

It must be admitted that some of the most prominent phenomena of neuralgia, in many cases at least, indicate the nervous centres, especially the spinal marrow and its branches, to be the original seats of disease. Some pathologists maintain that all neuralgic affections are dependent on primary spinal irritation. This is a simple and attractive doctrine, and hence has many advocates. In the early part of my professional life, I was captivated with it; and emboldened by the confidence inspired by high authority, as that of Teale, I treated every case of neuralgic character by cupping, counter-irritation, &c., over the spinal column, believing that every case presented more or less evidence of spinal affection. But as I became enlightened by experience, I was induced to conclude that, whilst it could not be denied that the nervous centres and branches may be the seat of primary disease, which may cause neuralgia in remote parts, it was too exclusive a view of its pathology, and that in order to the adoption of a rational, and successful treatment, it was necessary, in many instances, to extend our investigation beyond the points of manifestation, and the origins of the spinal nerves—with the view of discovering the ultimate cause, and of directing our remediate agents, in an especial manner, to its removal. The facts which led to this conclusion are the following: 1st. Many examples of neuralgia occur, in which, upon the most critical examination, there is an entire absence of those local signs which are considered to be characteristic of actual spinal disease. 2d. The utter inefficiency of the most potent local applications, in very many cases, where, apparently, the most unequivocal manifestations of actual spinal disease do exist. 3d. That, in numerous instances, patients after having been tormented, and nearly exhausted by cupping, blistering, tartar-emetic ointment, &c., and whom both physicians and friends have despaired of ever relieving, have often gradually recovered under the influence of alterants, mild aperients, and a general roborant course. 4th. That in a large majority of the severest cases of neuralgia which have ultimately proved fatal, autopsy has not disclosed the slightest appreciable lesion, which could account for the painful symptoms during life. In view of the above facts, I cannot doubt but that, in a large proportion of the very worst cases which come under our care, the local neuralgic affections are sympathetic of remote visceral irritations, which, through the medium of the spinal branches of the great sympathetic, morbidly impress the spinal marrow, and that the impression thus received being irradiated along its various trunks, is manifested by severe pain, or some disagreeable sensations, most generally towards the terminal-points of the spinal apparatus.

Unless we take this more comprehensive view of the pathology of neuralgia, I feel assured that, in but too many instances, we shall attack the shadow and not the substance of the insidious enemy with which we are contending, if we direct our chief efforts to those points on which its energies *appear* to be expended.

The facts which seem to prove the correctness of this view of the pathology of neuralgia are the following:

1st. The sympathy which is acknowledged by all medical men to exist between the viscera and the general nervous system. There are many familiar and striking illustrations of this connection. The numbness of the lower extremities from calculus in the kidney; the pain and numbness of the shoulder and arm from chronic hepatitis; the agonising headache from disordered stomach and liver, the severe pain in the head, back, and extremities so common in bilious and other fevers, accompanied with marked evidences of visceral derangement; the paroxysms of hysteria in adults, and the convulsions in children, from chylopoietic derangement; afford the most satisfactory evidence of the intimate connection between the various internal viscera and the nervous system.

2d. The undoubted dependence of various forms of paralysis on visceral diseases. The cases of paraplegia reported by Stanley, and proven to be dependent on diseased kidney; and those of Abernethy, dependent on hepatitis and enteritis, afford strong testimony in support of the important connection referred to.

3d. The utter inefficacy of all remedies applied to the spinal column, in cases of paralysis and neuralgia, connected with visceral diseases, whilst the symptoms of the former are often found to decrease, "pari passu," with the removal of the latter.

To conclude this summary view of the pathology of neuralgia, I would remark that from the light which we at present possess in connection with this intricate subject, we are forced to the conviction that, whilst it must be admitted that the nervous centres and trunks are susceptible of primary disease, the consequence, most generally, of mechanical injuries, of disordered circulation in the nervous envelops, or of disease in the bony casements, in, perhaps, much the largest proportion of cases of neuralgia which come under our notice, disorder or disease of some of the internal viscera will be found to be the "fons et origo" of this most afflicting malady.

My object, in the next place, will be to illustrate by a few interesting cases, selected from a number that have come under my observation, the above important pathological principle, which, if acknowledged by some, is far from being properly estimated by the profession at large, and the adoption of which would, in my opinion, conduce very much to the alleviation of human suffering.

Case 1st. The first case to which I shall refer, came under my notice in 1831, in Virgina, where I then resided; and as it presented some interesting features, I will give it somewhat in detail. The subject, Mrs. B., was a woman of delicate constitution, and aged about seventy. During my absence from home I was sent for to relieve her of what was supposed, by the family, to be an unusually severe attack of bilious colic, to which she was subject. Two other physicians of intelligence and skill were called in, and continued for several days to attend the case. Despairing, however, of being able to afford her any relief, they abandoned her to die. On my return home I was sent for again, and found her in the following condition: her pulse was very much sunk; her skin pale, shrunken and bathed in cold clammy sweat; there was great muscular prostration; she suffered intense pain and retraction of the abdomen; there was rigid constipation, and almost continued vomiting of fæcal matter, which, I was informed, had continued for several days. Here was a strongly marked case of intussusception. Having understood that all the usual remedies had been diligently applied, with but little relief, I apprehended that the case was hopeless, as I thought it highly probable that mortification had already taken place in the intestine. Being solicited, however, to do something for her, I gave her a very large anodyne, and some diffusible stimulants in the first place, and ordered a hot bath to be prepared. During the preparation of the bath, I made up twelve pills consisting of one drop of pure croton oil to the pill, and determined to administer one every two hours, until some decided effect should be produced. I also administered an enema, containing twenty grains of tartar-emetic, and about two drachms of laudanum, immediately after which she was placed in the hot bath. Under the combined influence of these means, a most fortunate and unexpected change took place in her case. The spasm of the intestine yielded; she discharged enormous amounts of peculiarly offensive matter from the bowels, consisting of hardened lumps; the vomiting greatly abated, and finally ceased; the abdominal pain and distention gradually gave way, leaving, however, considerable soreness. So great was the amendment, even in the course of twelve hours after I saw her, that I entertained a strong hope of her speedy recovery. Apprehending the occurrence of severe inflammatien, I continued the use of mild aperients, anodynes, and fomentations to the abdomen. These means, however, proved unavailing in warding off a severe attack of enteritic and peritoneal inflammation which again placed my patient in imminent danger. This was combatted by extensive cupping; calomel and opium, two grains of the former to one of the latter, every two hours; with occasional doses of castor-oil, and spirit of turpentine; hot mush poultices, and lastly, a large blister over the abdomen. Under this treatment the abdominal inflammation greatly subsided, but took on a chronic character. She now began to complain of uneasy sensations, such as numbness, pricking, soreness, and twitchings in her right leg, midway between her knee and ankle-joint. These sensations increased in intensity until there was permanent and severe pain, which was subject to occasional paroxysms of greatly increased violence. For the relief of the neuralgia every local remedy that could be thought of was applied; blisters above and below the seat of pain; various anodyne plasters; together with large anodynes internally, were used unavailingly. She continued to suffer most severely for several weeks, with but little mitigation. As there was not the slightest indication of spinal disease, and as the whole history of the case appeared to justify the opinion, I considered the neuralgia as sympathetic of the abdominal affection, and discontinuing the use of local remedies, I contented myself with the adoption of such means as were calculated to subdue the chronic inflammation. With this view, small doses of calomel and opium, mild aperients, and repeated counter-irritation were diligently made use of. Under this course of treatment, the abdominal inflammation disappeared, and with it, the neuralgic affection. Though the patient was now a good deal reduced in consequence of long continued severe pain, and irritative fever, I still hoped that she would ultimately recover. Soon after the disappearance of the neuralgic affection, the extremity below the seat of pain became discolored, assuming a dark livid appearance; it became unnaturally cold, insensible, and dwindled away almost to nothing. This condition I regarded as a modification of dry gangrene, and treated it with tonics, as mineral acids and quinine, opiates, wine, and nourishing diet. I proposed to amputate, assuring the family that it afforded the only reasonable ground of hope. One of the physicians who first attended the case being consulted, the operation was opposed upon the ground that, in all probability, the patient would sink under it, in consequence of her advanced age, and exhaustion from previous pain. The operation was not performed, and she gradually sank under the influence of irritative fever. My impression at the time was, and now is, that her life might have been saved by timely amputation. The above case presents several points of great interest: 1st. The successful termination of the case, so far as the intussusception was concerned, under apparently the most desperate circumstances. 2d. The cotemporaneous appearance and disappearance of the neuralgia and the abdominal inflammation, showing the relation of cause and effect. And 3d. The occurrence of dry gangrene, after the subsidence of the neuralgia.

Case 2d. This case came under my notice in 1835, in Maury county, Tennessee. The subject of it, Mrs. W., was aged about twenty-five, and was of good constitution. When she first consulted me, the most prominent symptoms of which she complained were referable to the chest. She had a violent cough, that was decidedly paroxysmal; there was constant pain in some part of the chest, which occasionally became intensely severe; the respiration was hurried and painful; the pulse was very little affected; the skin was of a natural softness, and temperature; and there was little or no expectoration.

There being no febrile symptoms, and no auscultatory signs to justify the opinion that there existed any serious thoracic affection, the most reasonable diagnosis that I could make was, that it was of a nervous or sympathetic character. With this view of its pathology, I administered mild aperients and alterants, for the purpose of rectifying the chylopoietic viscera; and at the same time made use of the most powerful anodynes, antispasmodics, and counter-irritation. This treatment produced but little mitigation of the cough, and pain in the chest. The pain which was at first confined to the chest, gradually extended to the shoulder, down the arm, and finally settled in the wrist and hand, in the form of the most acute neuralgia. For the relief of this, every remedy both general and local, that is usually adopted, was fairly tried. The most powerful internal anodynes were administered; and topical applications, as cupping, counter-irritants, and anodyne embrocations were made from the origins of the spinal nerves, which go to form the axillary plexus, down to their digital extremities; but all proved of little avail. Having, as I thought, exhausted all the known resources of medicine, I was strongly tempted to resort to surgical aid. It occurred to me that strong pressure either with the tourniquet or well adjusted bandage might be of service. This means, however, affording but little relief, I ventured to propose a division of the nerve, but the patient would not consent. I now nearly despaired of ever being able to relieve my patient, and discontinued the use of almost all the remedies, advising her to rub her arm and wrist occasionally with some anodyne and stimulant liniments, and to keep the painful parts well wrapped up. Soon after giving this advice, a most fortunate circumstance occurred, which, at once, developed the cause of her singular and afflicting case, and which afforded almost instantaneous relief. During an unusually severe attack of cough, she ejected either from the lungs, or larynx, an exceedingly irregular and rough calcarious mass, about the size of a common bean. This was followed by a rapid abatement of the pectoral symptoms, and the local neuralgia; and her general health soon became as good as ever.

In full view of the whole history of the above interesting case, I could not doubt the dependence of the violent paroxysmal cough, and the acute pains of the chest, on the mechanical irritation arising from the presence of the body referred to; nor did it appear less obvious, that the neuralgic affection of the arm and wrist was sympathetic of pulmonary or laryngeal irritation.

Case 3d. This case presented itself to my notice in 1839, in the State of Alabama. The subject, Mrs. G., was aged about twenty-five; and of rather a delicate, nervous constitution. This case was characterised by its great severity, and unusual number of anomalous symptoms, the detail of which would occupy the compass of an ordinary communication. As I design to be as brief as possible, I will content myself with giving merely a general outline of the symptoms, and course of treatment pursued.

When I first saw her, she presented obvious indications of general nervous depression; her complexion was pale and sallow; her appetite slight and capricious; her muscular strength was much diminished; and there was a general sense of numbness, pricking, twitching, and of the passage of cold water, principally in the lower extremities; and there was unusual mental depression. For the above symptoms I administered mild aperients, and advised the use of strong liniments, and friction over the extremities, together with the precipitated carbonate of iron. These means afforded but little relief. The uneasy sensations, especially in the lower extremities, continued to increase in intensity, until she suffered the most acute pain in the back, and along the whole course of the sciatic nerve and its branches. The pain at length became paroxysmal, and starting in the back, would in an instant radiate along all the trunks proceeding from the lumbar and sacral portions of the spinal marrow; and would often come on with so much violence that it produced a general convulsive movement of the body. She next suffered severe pain in the chest, accompanied with dyspnœa, and often inordinate and irregular action of the heart. Severe pains then occurred in the upper extremities, attended by great numbness, and loss of the sense of touch, and power to grasp, so much so, indeed, that it was with the utmost difficulty that she could hold any small body, as a needle or pin, in her fingers. At this stage of her case, the chief source of her suffering was the most severe lancinating darting pain in the face and scalp, occupying but one side at a time, but rapidly shifting from side to side. She frequently complained of a very curious and uncommon sensation about the brain, which, to use her own language, seemed, just before each severe paroxysm of pain, to rise up and expand, so as to become much too large for the casement which enclosed it; hence arose a most distressing sense of oppression and stricture about the head. There was morbid sensibility of the eye and ear, to such a degree that strong light or loud sounds greatly aggravated her sufferings. For some time she had chills which seemed to observe a regular periodicity; and so great was her depression during the paroxysms, that the family, and her uncle, an intelligent physician, who frequently saw her with me, thought that she could not possibly survive another.

During the whole course of this case there was another prominent symptom, which I have designedly reserved for the last of the catalogue of phenomena, as it seemed very distinctly to point to the spinal column as the immediate seat of the disease; this was exquisite tenderness at different points of the spinal column, from the lower cervical to the sacral vertebræ, when pressure was made. Severe pains could be caused in distant parts by even moderate pressure on the painful portions of the spine. Besides the tenderness just referred to, there was a general sense of soreness in the skin and flesh over nearly the whole body and extremities. The advocates of the doctrine which ascribes most, if not all of the neuralgic affections to immediate spinal disease, would pronounce the last mentioned symptoms as pathognomic of such a condition. They are said, by the best authorities, to be universally present in cases of inflammation of the envelopes of the spinal chord. After a careful investigation of the case, I was led to the conclusion that all of the symptoms which I have detailed, were referable to some diseased condition of the spinal marrow. With this view of its pathology, I made use of cupping along the whole course of the spine, but chiefly over the painful points; using the scarificator and small tumblers I succeeded in drawing a considerable quantity of blood. The cupping was often repeated, and after their application, blisters, or tartar-emetic ointment were applied. In addition to the above treatment, large doses of opium, or sulphate of morphia were occasionally all ministered to mitigate the pain; and mild purgative pills, as the compound colocynth pill, were given to regulate the bowels. Every time the cups were applied there was a great palliation of all the symptoms, which caused my patient to desire its frequent repetition, and my willingness to acquiesce in her wish. But unfortunately, this temporary mitigation was invariably succeeded by an increased aggravation; the patient becoming much weakened, and obviously worse. To obviate this effect, and with the view of making a strong new impression on the nervous system, I administered the sulphate of quinine and the precipitated carbonate of iron, alternately, in as full doses as she could bear. These disturbed her head and stomach very much, and were, therefore, discontinued.

At this stage of the case my intelligent friend Dr. Helms, of Tuscumbia, Alabama, was called in consultation. I gave him a full history of the case, and the course of treatment pursued. He remarked that the symptoms under which she had labored, and, indeed, which she then suffered, seemed to indicate the existence of spinal affection, but suggested that, as the treatment which had been the most appropriate that could have been adopted, upon the supposition of the existence of such a condition, was not only proving unavailing, but rather injurious, we should direct our attention to some other point, with the view of discovering the true pathology; and intimated that morbid sensibility of the gastric nerves, and hepatic disorder might be the origin of the afflicting nervous affections. Having failed with the most potent remedies addressed to the spine and to the general nervous system, and readily conceiving how the latter conditions, supposing them to exist, could produce the phenomena exhibited in the case, I unhesitatingly assented to a change of treatment, and to the adoption of means calculated to restore the chylopoietic viscera to a healthy condition; and at the same time to impart tone and vigour to the general system. Having agreed, in consultation, on the general indications to be pursued, I adopted the following plan of treatment. In order to improve the biliary secretion, to allay the morbid sensibility of the stomach, and to keep the bowels very gently evacuated, I combined one drachm each of blue-mass, rhubarb, and ext. hyosciamus; and divided the mass into thirty-six equal pills, of which I administered one or two every four or six hours, according to circumstances. When the pain was very severe, and there was great restlessness, I resorted to the black drop, in full doses, with an excellent effect. For the purpose of imparting tone to the general system, the sulphate of quinine, precipitated carbonate and the muriated tincture of iron were alternately tried, together with port-wine and cordial diet. The quinine and preparations of iron disturbed the head and stomach to such a degree, that they were dispensed with, and the oxide of bismuth alone, or combined with ext. hyosciamus, in the proportions of five grains of the former to three of the latter, in the form of a pill, was continued, three times a day. Hoping that the tonic and revellent influence of exercise, and change of air and scene, might exert an important impression upon the general nervous system, by modifying its sensibility, which appeared to be excessively exalted, and thus break up the chain of morbid associations, I determined to try the effect of carriage exercise. My patient, when much too weak to walk, was lifted into a carriage morning and evening, and the distance was increased a little every time. During the intervals between the rides, I required the room to be more freely ventilated than it had been; and requested her to occupy her bed much less than she had done; and caused extensive and forcible frictions to be made over the whole surface. Under the foregoing treatment, which, with some unimportant modifications, was continued for two or three weeks, to my great gratification, my patient continued to amend from the time of its adoption; the chylopoietic viscera becoming restored to a healthy condition; the nervous system reduced to its normal state of sensibility; the severe pains and all other unpleasant sensations gradually disappeared, and her general health soon became better than it had been for several years. I received a letter a few weeks ago from my patient's husband, now a resident of Mississippi, in which he stated that she had enjoyed excellent health ever since her recovery from her severe attack of 1839.

The above case was the most interesting that I ever met with. 1st. Because the neuralgic affections were the most extensive, involving, at different periods during its course, almost every part of the nervous system, from the great centres, to their terminal ramifications. 2d. Because it was attended with an unusual number of anomalous symptoms, only a small part of which I have enumerated. 3d. Because all of the most prominent features of it indicated the existence of serious spinal affection; whilst at the same time the remedies directed to its removal obviously aggravated the symptoms. 4th. Because the successful termination of the case was, in my opinion, unquestionably attributable to the use of those means which were administered with the view of removing the supposed visceral disorders.

Taking an impartial view of the whole history of this case, and the effects of treatment, I cannot but conclude that the exquisite tenderness on pressure, along the course of the spinal column, the general soreness of the flesh, and all of the more prominent neuralgic affections, were dependent on gastric and hepatic disorder which morbidly impressed the spinal marrow and trunks, through the medium of the great sympathetic.

Case 4th. This case came under my notice in 1841. The subject of it, Miss G., a resident of Giles county, Tennessee, was aged about twenty, and naturally of rather robust constitution. The history of her case previous to the time at which I first saw her, was substantially as follows: In the fall of 1839, she was attacked with a severe form of fever, which from the information I received from the family, I suppose was of the remittent bilious character, attended with strong evidences of muco-gastritis and enteritis, and of cerebral irritation. The case was attended by one of the charlatan tribe, who, advocating the propriety of keeping the alimentary canal well washed out, so as to secure a *clean house*, treated it mostly on the purgative plan, administering almost throughout its whole course, if correctly informed, the most drastic cathartics, as enormous doses of aloes, &c. Nature triumphed in a good degree over this *rude* assault; and the patient convalesced very slowly, and was *partially* restored to health. The digestive organs continued for a considerable time very much enfeebled; and the menstrual function was totally suppressed. About the 1st of December, she was attacked with violent pains in her back and extremities, especially the lower; for the relief of which, as informed by the family, active cathartics, and very large doses of opium were administered by her original attendant. As all of her symptoms grew rapidly worse, Dr. F., a practitioner of intelligence and experience, was called in. He regarded the case as one of common hysteria, dependent on uterine derangement, and treated it with purgatives, anodynes, antispasmodics, and emmenagogues. Under this treatment the patient appeared to improve at first, but was subject to repeated relapses, during which the symptoms were greatly aggravated. Dr. F. being at the distance of thirteen miles, and being at the time much engaged, did not visit her very often. Another physician of intelligence, Dr. D., who resided in the neighborhood, was called in. He pronounced the case to be one of acute rheumatism, and treated it with free bloodletting, purgatives, and opiates. As the case grew worse under this treatment, Dr. F. was again requested to attend, and after visiting two or three times, and adopting the same plan of treatment he employed when he first saw her, with the addition of cupping to the spine, and the use of quinine, he desired that I should be called in consultation. I visited the case with Dr. F. on the 11th of January, 1841; and found the patient in the following condition. The complexion was remarkably pale and sallow; the whole surface was moist, cool and flaccid; the pulse was quick and irritable, and of moderate volume; the tongue was moist, rather pale, and coated; the bowels were constipated. She complained of almost constant pain in the lumbar and sacral portions of the back, about the os coccygis, and along the whole course of the sciatic nerve and its branches. There was very great tenderness on pressure, at various points of the spinal column. Soon after entering the patient's room, she was seized with a most violent paroxysm of pain which, starting, as she said, *in the small of her back*, seemed to fly with the rapidity of lightning throughout the whole spinal apparatus; causing her to scream out loudly, and to writhe upon the bed. Such severe pain could be generally produced by firm pressure over the tender points of the spine. Upon inquiry, I learned that she had had these paroxysms of acute lancinating pain for a considerable time; that at first, they came on at somewhat distant intervals, and, at length, occurred as often as five or six times a day. In addition to the foregoing symptoms, she often suffered acute lancinating pains in her chest, with difficult respiration, evidently of the nature of pleurodynia, which appeared and disappeared very suddenly. After the severe paroxysms passed off, there was considerable languor and debility; and during the whole interval, the most disagreeable sensations of numbness, pricking, and twitching, especially in the lower extremities, were experienced. She scarcely slept at all, unless under the influence of the most powerful anodynes. During the whole course of her afflicting illness, her mind was not at all affected, being always conscious, when awake, of every thing that was said and done.

I felt no hesitation in pronouncing this a decidedly marked case of neuralgia, dependent, perhaps, on spinal irritation, but most probably on functional derangement of the chylopoietic viscera, and especially of the uterus, the sequelae of a protracted and badly managed case of fever. With this view of its pathology, I advised the repeated application of cups, along the whole course of the spine, to be followed by blisters or tartar-emetic ointment, by which severe counter-irritation was kept up for some time; together with full anodynes to quiet the nervous system; quinine and precipitated carbonate of iron, to invigorate the general system, but chiefly with the view of modifying nervous sensibility, by virtue of the new impression they are capable of making; and purgatives to keep the bowels well evacuated. The above plan of treatment with some little variation was pursued diligently for several weeks, during which time, Dr. F. and myself continued to visit her alternately. It is worthy of remark that, although the signs which usually indicate the existence of spinal irritation, were present in a marked degree, all the topical remedies as cupping, blisters, and tartar-emetic ointment, obviously aggravated all the symptoms of the case. The first effect was that of palliation, but invariably the brief calm thus procured, was followed by increased violence of the paroxysms.

The anodynes and the tonics appeared to be well borne, but produced only a temporary effect. The patient continued to endure her painful situation with very little variation, excepting that, occasionally, she enjoyed an exemption from her painful paroxysms for two or three days together, until the 7th of April, when Dr. F. being compelled to abandon the case in consequence of numerous engagements, I was requested to take the entire charge of it. I visited her on the 7th, and spent the night with her for the purpose of making a critical examination of her case. Soon after I entered the house she was seized with an unusal attack of pain that continued a considerable time. I was informed that she had not slept more than one hour for several days. She was exceedingly pale and debilitated. As all the remedies which we had used, and they too the most potent, especially the topical, had proved unavailing; I concluded that there must be some visceral derangement that was the source of the evil, and which most probably had not yet been reached. A careful examination of the case convinced me that the stomach and liver were very much disordered, probably in a good degree the effect of excessive use of opium, which had been liberally used from the commencement of the case; and that the uterus was in a state of great sanguineous engorgement, and perhaps of chronic inflammation, as the patient complained of a constant sense of weight and pressure low down in the pelvis, also of pain about the pubic region, and soreness about the neck of the uterus. The case now presented a new aspect; and it occurred to me that, if the congestion and irritation of the uterus could be removed, and the digestive organs placed in a healthy condition, the whole chain of morbid phenomena would be broken up. The next important question for determination was, what means will best effect these desirable objects. After some deliberation, I determined to adopt the following simple course. In the first place I inserted a large seton low down in the back, and applied a blister on the inside of each thigh, directing them to be repeatedly applied. I ordered the warm hip-bath to be made use of twice or three times a day; and the tepid shower bath twice a day. To improve the biliary secretion, and act gently on the bowels, I gave every second night one or two pills of equal proportions of blue-mass, rhubarb and aloes. I occasionally administered a full anodyne, as black drop, or Dover's powder, at night, to secure rest. The precipitated carbonate of iron was continued for some time, as a tonic. I urged her to exert herself, and rise out of bed, and exercise first in the house, then in the open air.

I was exceedingly gratified with the results of the above plan of treatment. I know not to what part the good effects were ascribable, but I do know that from the time of its adoption my patient began to improve; the paroxysms became rapidly weaker, and the intervals longer, until they disappeared entirely, which was about the 30th of April, after which time I administered no more medicine, excepting some gentle aperient pills to regulate the bowels. After this, in compliance with my urgent advice, she took a great deal of horse exercise, which, invigorating the digestive organs, conduced to the establishment of the menstrual secretion, soon after which her general health became as good as ever, and continues to be so.

An impartial review of the whole history of the foregoing interesting case, and the effects of treatment, affords conclusive evidence to my mind, that all the phenomena which characterised it, were sympathetic of gastric, hepatic, and especially uterine derangement.

I could enumerate other cases which I could select from a number that I considered worthy of being noted, but I deem it unnecessary, as those already cited, are altogether sufficient to illustrate the important pathological principle which it has been my object to establish, namely, that most of the neuralgic affections that present themselves to our notice, are dependent on visceral derangements, the morbid action of which, being communicated through the spinal branches of the great sympathetic, to the spinal marrow and trunks, induces that exaltation of nervous sensibility and morbid impressibility which exist, in so high a degree, in this truly afflicting class of human maladies.

